# The mediating role of cognitive test anxiety on the relationship between academic procrastination and subjective wellbeing and academic performance

**DOI:** 10.3389/fpubh.2024.1336002

**Published:** 2024-06-10

**Authors:** Ion Albulescu, Adrian-Vicenţiu Labar, Adriana-Denisa Manea, Cristian Stan

**Affiliations:** ^1^Faculty of Psychology and Educational Sciences, Babeş-Bolyai University, Cluj-Napoca, Romania; ^2^Faculty of Psychology and Educational Sciences, Alexandru Ioan Cuza University of Iaşi, Iaşi, Romania

**Keywords:** academic procrastination, anxiety, academic performance, subjective wellbeing, education, self-regulation

## Abstract

**Background:**

Promoting wellness as a predictor of sustainable development empowers schools to model healthy behavior. The multiple interactions in real and virtual environments that today's youth are subjected to force schools to explore effective educational strategies to provide a quality education for students and their families.

**Purpose:**

This study examines the relationship between academic procrastination, assessment anxiety, subjective wellbeing, and academic performance.

**Methods:**

A convenience sample of 322 undergraduate students () was used, and questionnaires were administered to students measuring academic procrastination, cognitive test anxiety, and subjective wellbeing. For the same target group, the level of academic performance was identified using personal reports. The questionnaires were administered between May and June 2023 in an online format. For the data analysis, we applied correlational analysis and path analysis using.

**Results:**

Both test anxiety and academic procrastination negatively correlate with performance and subjective wellbeing, leading to decreased performance and subjective wellbeing. Procrastination correlates positively with test anxiety. Cognitive test anxiety partially mediated the relationship between academic procrastination and subjective wellbeing and fully mediated the relationship between academic procrastination and academic performance. Thus, high procrastination leads to decreased performance and subjective wellbeing both directly and indirectly through increased test anxiety, leading to decreased performance and subjective wellbeing.

**Significance/discussions:**

As a result of theoretical and practical investigations, it emerges that joint action of educational actors is required in the generation of effective educational strategies for the prevention and control of procrastination and evaluation anxiety, given the fact that both a high level of procrastination as well as assessment anxiety led to the decrease of students' wellbeing, to the registration of low academic performances. In the long term, disruptive behavior (procrastination and anxious behavior) could generate low social and professional performance, which is a research question for a future longitudinal study.

## 1 Introduction

The issue of procrastination and assessment anxiety concerns more and more specialists in the educational field, given their negative effect on the desirable development of students. According to some studies on procrastination understood as a postponement of solving various tasks (1, 2), the need for educational interventions to facilitate/determine behavioral self-regulation was highlighted (behavior derived from a mix of cognitive, affective, and motivational abilities). Also, studies on assessment anxiety classified as social phobia (3), fearful emotions and somatic reactions, their negative feelings continue to increase until they begin to experience a panic attack (4) they highlighted the importance of self-regulated learning. We consider the negative effects and subsequent implications of assessment anxiety and procrastination regardless of gender or type of procrastination (5–8). Considering the sources of assessment anxiety which mainly include students' uncertainty about the nature/content of the requirements formulated, the expected results as well as their ability to satisfy these requirements (9) it is necessary to intervene on the control of metacognitive beliefs, especially since it is known that repetitive negative thinking and anticipatory exaggeration of the consequences of failure is one of the defining characteristics of evaluation anxiety (10). Also, we consider the negative effect of procrastination/postponement on academic performance, stress, wellbeing, mental health (10–19). The negative and low association between procrastination and academic performance is highlighted in several studies, with the emphasis that procrastination is sometimes harmful, sometimes harmless, but certainly useless (20). At the same time, it confirms the negative relationship between procrastination and academic performance (21), it should be emphasized that procrastination has the effect of registering a reduced degree of wellbeing (22, 23), with the mention that it is necessary to take into account cultural education regarding the negative effects of procrastination on wellbeing (12), which implies because active procrastination can have a beneficial effect on psychological wellbeing (24–26). Moreover, psychological wellbeing is a negative predictor of procrastination (27). Procrastination increases stress in young adulthood, being more common in men than in women (28–30). It should be noted that negative emotions are associated with a lower level of psychological wellbeing (18). Of course, some gaps are reported regarding the causes of procrastination, such as emotions or other antecedents generating situational/contextual and/or dispositional procrastination (31). Therefore, preventive strategies should be considered in order to overcome procrastination, which affects academic results, satisfaction, and wellbeing (32), especially since test anxiety and time management have also been associated with academic procrastination (33). So, time management and psychological effort in the multidimensional approach to perfectionism must be considered when the goal is to reduce procrastination (34, 35). Procrastination and assessment anxiety are significantly related to low levels of self-efficacy for self-regulation (10, 36). There are factors that cause anxiety among students correlated with academic performance, namely peer acceptance, uncertainty about the nature of expected demands, and their ability to meet these demands (9), doubts about their intellectual capacities, their time management capacities (37, 38), insufficiently defined institutional standards for marking quality items, concerns about the fairness of certain types and assessment tools (e.g., across studies undertaken, students reported that oral presentations and high-stakes written assignments caused them more anxiety) (39). The range of anxiety-generating factors also includes the parenting style faced by the student (40–42), the negative, repetitive thinking about the consequences of failure that can lead to self-judgment and over-identification (10, 43). It should be emphasized that all these factors are added to those generated by learning in open spaces, as a result of online education (44). Self-assessment of the level of procrastination and active involvement in school-related tasks are rated as good/valid and available solutions for students to reduce/diminish the negative influence of procrastination in their further development, including professional (45–47). Other educational intervention strategies for reducing procrastination are related to increasing organizational group pressure in the context of a positive learning environment (48), developing cognitive and non-cognitive skills, increasing self-efficacy, self-control (48–52), increasing social resilience, given that social anxiety partially mediates the relationship between resilience and procrastination (53). Because there is a positive association between fear of failure and academic procrastination on the one hand and a negative association between self-regulation subscales and academic procrastination on the other, it is necessary to intervene by developing self-regulation strategies and inhibiting/neutralizing fear of failure, balanced distribution of the workload, practicing learning in stimulating environments (11, 54, 55). According to the latest studies on the role of context in vulnerability to procrastination, we understand the need to focus attention on those ongoing stressful circumstances that could slow down the self-regulation process and consequently increase vulnerability to procrastination (56), especially as the irrational nature of this habit of delaying the start or completion of an action/task is known (57), although the repercussions of the delay are realized (20, 58). Moreover, procrastinating behavior persists in many procrastinators who wish to continue having such experiences (59) and those who seek help for self-regulation problems inherent in procrastination do so more because of feelings of shame associated with procrastination (60). Thus, procrastination is a significant negative predictor of subjective wellbeing, which suggests to us that difficulty in completing tasks on time is an indicator of decreased mental health and decreased life satisfaction in an individual, with procrastination being a predictor of subjective wellbeing (25, 27), especially when we talk about active, controlled, directed procrastination (24).

Therefore, adding to the aforementioned findings, we bring forth the idea that individuals' academic procrastination might be a significant predictor of subjective wellbeing and academic achievement. More specifically, we hypothesized that:

H1. Academic procrastination and cognitive test anxiety are negatively related to subjective wellbeing and academic achievement.

H2. Academic procrastination is positively related to cognitive test anxiety.

H3. Cognitive test anxiety mediates the negative relationship between academic procrastination and subjective wellbeing.

H4. Cognitive test anxiety mediates the negative relationship between academic procrastination and academic achievement.

The concern of the authors of the study as trainers in education is to achieve high academic performance by students and ensure a subjective wellbeing makes the research focus on highlighting the causes that contribute to the decrease in academic performance.

The study contributes to highlighting the relationship between procrastination, and subjective wellbeing and school performance, by analyzing the explanatory role of cognitive test anxiety on the relationship between procrastination and subjective wellbeing, and school performance, and consequently to indicate the directions of educational action in order to achieve the school health necessary for individual and social progress.

## 2 Materials and methods

### 2.1 Participants and procedure

The convenience sample comprised of 322 undergraduate students (66.5% women and 33.5% men) aged 18 to 27 years (*M* = 22.53, *SD* = 6.60). The sample of subjects was selected from the students of the specializations: Pedagogy, Teachers for primary and preschool education at Babes-Bolyai University, Cluj-Napoca After providing informed consent, participants completed the questionnaire online. The students were informed that their participation was voluntary and that the answers were confidential.

### 2.2 Research design

A cross-sectional study design is considered to be optimum, since it investigates the relationships between academic procrastination, cognitive test anxiety, and subjective wellbeing at a certain time.

### 2.3 Measures

*Academic procrastination*: Academic procrastination was assessed by five five-point Likert scale items (1 = strongly disagree; 5 = strongly agree) adapted from the Academic Procrastination Scale-Short Form (61). The English version was translated into Romanian and then back translation into English was used in order to ensure semantic equivalence. A sample item is “I put off projects until the last minute.” Cronbach's alpha is 0.88. Average scores were computed, with higher scores reflecting more academic procrastination. In order to verify the factorial validity of the scale, we used confirmatory factor analysis (CFA). For the model fit, we applied the maximum-likelihood estimation and reported the following fit indexes: Comparative Fit Index (CFI), Tucker-Lewis Index (TLI), Standardized Root Mean Square Residual (SRMR), and Root Mean Square Error of Approximation (RMSEA). The indices of the confirmatory factor analysis revealed an acceptable to excellent fit of the one-factor solution as proposed by the author (with one pair of errors correlated): χ^2^(4) = 8.88, *p* > 0.05, : χ^2^/ df = 2.22; CFI = 0.99; TLI = 0.98; SRMR = 0.02; RMSEA = 0.06, 90% CI: [0.00, 0.11].

*Cognitive test anxiety:* The cognitive test anxiety was assessed using the Cognitive Test Anxiety Scale-Short Form (CTAS-SF) (62). The CTAS-SF is a 17-item revision to the original CTAS that uses a four-point Likert scale (1 = not at all like me, 4 = very much like me) and was validated on the Romanian population (40). A sample item is “My mind goes blank when I am pressured for an answer on a test.” Cronbach's alpha is 0.93. Average scores were computed, with higher scores reflecting a more cognitive test anxiety.

*Subjective wellbeing:* Subjective wellbeing was assessed using the Satisfaction with Life Scale (63). SWLS is a 5-item scale that used a seven-point Likert scale (1 = strongly disagree, 7 = strongly agree) and was validated on the Romanian population (64). A sample item is: “In most ways, my life is close to my ideal.” Cronbach's alpha is 0.83. Average scores were computed, with higher scores reflecting a greater life satisfaction.

*Academic performance:* Students' academic performance was measured by their anterior semestrial grade. Students were asked to report their anterior semestrial grade as part of a brief demographic questionnaire. The Romanian grading scale ranges from 1 (poor) to 10 (outstanding).

### 2.4 Data analyses

The data analyses were conducted using IBM SPSS and AMOS version 24.0. First, we calculated descriptive statistics, Cronbach's alpha, and correlations between measures. Then, path analysis using maximum likelihood estimation was conducted to examine our research hypotheses regarding the mediating effect of cognitive test anxiety on the relations between academic procrastination, and subjective wellbeing and academic performance. To evaluate the overall fit of the model to the data, the following elements were calculated in the present study: chi-square statistic (χ^2^), χ^2^/df ratio, Standardized Root Mean Square Residual (SRMR), Root Mean Square Error of Approximation (RMSEA), Comparative Fit Index (CFI), and Tucker-Lewis Index (TLI) (65, 66). Goodness-of-fit criteria were used in the current study that acknowledged the potential for acceptable (χ^2^/df ratio < 3, CFI and TLI > 0.90, SRMR < 0.10, RMSEA < 0.08) and excellent fit (χ^2^/df ratio < 2, CFI and TLI > 0.95, SRMR < 0.08, RMSEA < 0.05). For mediation analyses, we used bootstrapping with 5000 bootstrap resamples to examine indirect effects in mediation models. Confidence intervals that do not contain zero indicate a significant indirect effect (mediation).

## 3 Results

### 3.1 Preliminary analyses

Descriptive statistics are reported in Table 1. As control variable, age was significantly positively correlated with subjective wellbeing (*r* = 0.16, *p* < 0.01), academic performance (*r* = 0.21, *p* < 0.001), cognitive test anxiety (*r* = −0.12, *p* < 0.05), and academic procrastination (*r* = −0.23, *p* < 0.001). An independent sample *t*-test was computed to analyze if there are gender differences regarding the main variables of the study. Results show that women have higher scores than men at subjective wellbeing [*t* (320) = 3.87, *p* < 0.001], academic performance [*t* (320) = 3.41, *p* < 0.001], cognitive test anxiety [*t* (320) = 2.51, *p* < 0.05], and academic procrastination [*t* (320) = 3.08, *p* < 0.01].

**Table 1:** Correlations among the main study variables.

**Variables**	***M***	***SD***	**Min**.	**Max**.	**1**	**2**	**3**
1. Academic procrastination	2.50	1.05	1	5			
2. Cognitive test anxiety	2.35	0.71	1	4	0.27^*^		
3. Subjective wellbeing	4.97	1.17	1.2	7	−0.22^*^	−0.19^*^	
4. Academic performance	8.45	0.92	5	10	−0.19^*^	−0.20^*^	0.03

*N* = *322*. ^*^p < 0.001.

### 3.2 Associations among main study variables

Zero-order correlations among variables are reported in Table 1. Academic procrastination was significantly negatively associated with subjective wellbeing (*r* = −0.22, *p* < 0.001) and academic performance (*r* = −0.19, *p* < 0.001) (H1), whereas significantly positively with cognitive test anxiety (*r* = 0.27, *p* < 0.001) (H2). Cognitive test anxiety was also significantly negatively correlated with subjective wellbeing (*r* = −0.19, *p* < 0.001) and academic performance (*r* = −0.20, *p* < 0.001) (H3). Hypotheses H1, H2, and H3 are therefore supported by our results.

### 3.3 Path analysis testing the mediation role of cognitive test anxiety

**Figure 1:**
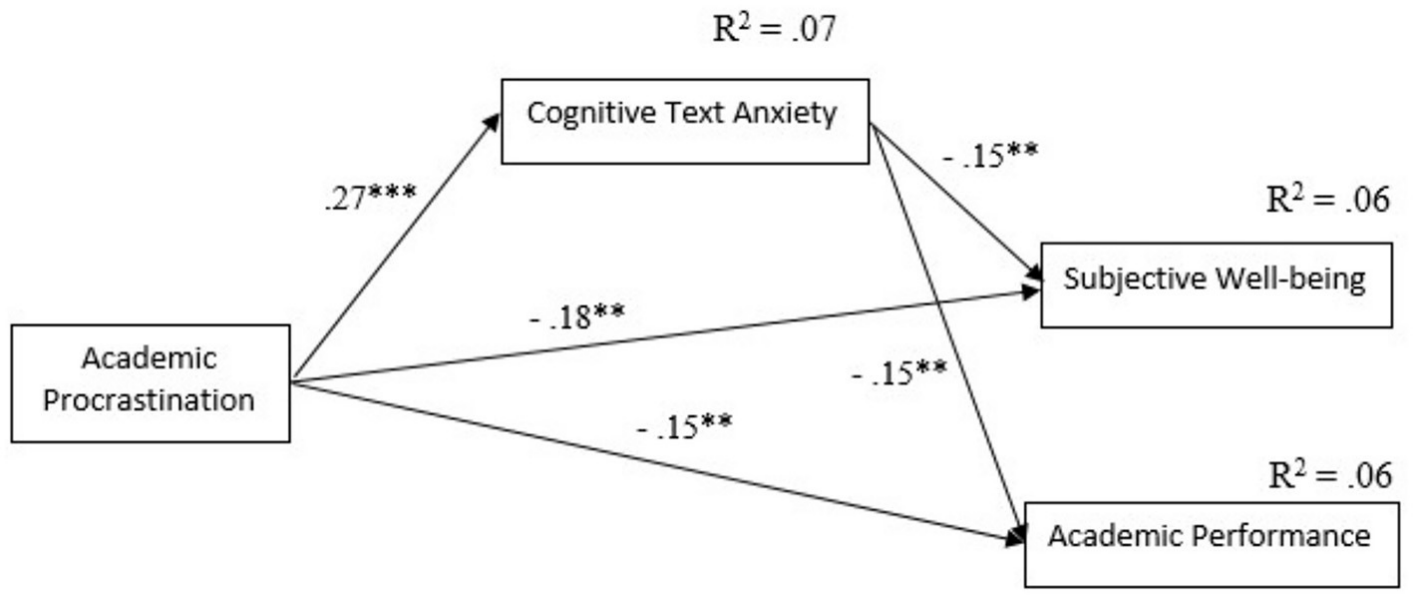
Path analysis testing the mediating role of cognitive test anxiety in the relations between academic procrastination and subjective wellbeing and academic performance. Standardized coefficients were reported. ^**^p < 0.01. ^***^p < 0.001.

In order to test our mediation hypothesis, a path analysis was conducted with academic procrastination as an independent variable, cognitive test anxiety as a mediator variable, and subjective wellbeing and academic performance as the dependent variables. The proposed mediation model (see Figure 1) had a good to perfect fit to the data (χ^2^ (1) = 0.507, *p* > 0.05, TLI = 1.05, CFI = 1.00, RMSEA = 0.00, 95% CI [0.00, 0.13], SRMR = 0.02).

The results of path analysis (Figure 1) showed that academic procrastination positively predicted cognitive test anxiety (β = 0.27, *p* < 0.001). Further, cognitive test anxiety negatively predicted both subjective wellbeing (β = −0.15, *p* < 0.01) and academic performance (β = −0.15, *p* < 0.01). Academic procrastination negatively predicted subjective wellbeing (β = −0.18, *p* < 0.01) and academic performance (β = −0.15, *p* < 0.01). The model explained 6 % variance of subjective wellbeing and 6 % variance of academic achievement.

The results of mediation analyses are presented in Table 2. The indirect effect of academic procrastination on subjective wellbeing (−0.040, 95 % CI [−0.083, −0.008]), and academic performance (−0.042, 95 % CI [−0.090, −0.011]) through cognitive test anxiety was significant. The direct effect of academic procrastination on subjective wellbeing was significant, and also the direct effect of academic procrastination on academic performance was significant. Therefore, hypothesis H4 was supported as cognitive test anxiety partially mediated the relation between academic procrastination and subjective wellbeing and between academic procrastination and academic performance.

**Table 2:** Standardized estimates of the direct, total and indirect effects of academic procrastination on subjective wellbeing and academic achievement through cognitive test anxiety.

**Predictor**	**Mediator**	**Dependent variable**	**Direct effect**	**Total effect**	**Indirect effect [95% CI]**
AP	CTA	SWB	−0.176^**^	−0.216^***^	−0.040^*^ [−0.083, −0.008]
AP	CTA	AP	−0.149^*^	−0.191^**^	−0.042^**^ [−0.090, −0.011]

^*^p < 0.05; ^**^p < 0.01; ^***^p < 0.001; results based on 5000 bootstrap samples; CI, bias-corrected 95% confidence interval for the indirect effects; AP, Academic procrastination; CTA, Cognitive test anxiety; SWB, Subjective wellbeing; AP, Academic performance.

## 4 Discussion

The recorded results are in agreement with some of the studies that addressed the issue of procrastination, evaluation anxiety, and, respectively, wellbeing. Thus, procrastination correlates negatively with student performance; in other words, school performance is negatively affected by procrastination and procrastination behavior (7, 23). At the same time, procrastination also correlates negatively with subjective wellbeing (7, 16, 29, 32, 54). Of course, it must be stated that in the interpretation of the results, we associated procrastination with passive procrastination, conceptually making the difference between active and passive procrastination, given the fact that active procrastination can be a sign of psychological wellbeing, of wellbeing (24), we can thus consider that some form of procrastination does indeed improve student performance and wellbeing (25). Also, it should be emphasized that the study highlighted the existence of gender differences in terms of the study variables, in the sense that women develop a procrastinating and anxious behavior toward evaluation higher than men on the one hand, but also academic performance and wellbeing taller than men, on the other hand. At the same time, the fact that the age of the subjects correlated positively with two variables of the study (subjective wellbeing, and academic performance) and negatively with the other two study variables (cognitive test anxiety, and academic procrastination) indicates that in adolescence, and especially in girls, attention should be focused on managing anxiety evaluation and procrastination. The study's results confirm, in agreement with other studies (19, 27), that procrastination correlates positively with test anxiety, while test anxiety correlates negatively with academic performance (43). So, given the negative effect of procrastination/ postponement on academic performance and wellbeing (5, 11–19) academic procrastination must be discouraged by building positive, collaborative, and effective learning environments (67). Likewise, the effects of procrastination on wellbeing could be modified by developing self-regulation mechanisms (11, 12), based on educational programs (8), even more so with a moderately significant negative relationship was found between self-regulation skills and students' test anxiety levels (36, 47). Levels of anxiety and procrastination were significantly related to low levels of self-efficacy for self-regulation (19), and our study, consistent with other studies, also showed a negative correlation between test anxiety and performance (40) and subjective wellbeing. Also, the study highlighted the existence of a partial mediating effect of test anxiety on the relationship between procrastination and subjective wellbeing and a fully mediating effect of test anxiety on the relationship between procrastination and performance; in other words, increased stress causes increased procrastination among students and vice versa (14). To develop self-regulatory skills and manage student procrastination, it is recommended to consider providing mentoring/guidance by career education specialists (45). In the case of online learning, self-regulation is achieved with increased difficulty, the risk of procrastination being greater, given the multiple variables, the learner faces: task strategy, mood adjustment, self-evaluation, environment structure, time management, and help-seeking (68). For a sustainable education, the lines of action derived from the conducted study aim to achieve wellbeing by reducing and controlling procrastination and evaluation anxiety. Reducing the stress generated by assessment anxiety can be achieved through a better organization of learning activities (active-participative, collaborative, and connectivist approach), effective time management, and emotional management of disruptive situations that generate academic procrastination.

The fact that our study showed that cognitive test anxiety partially mediates the negative relationship between academic procrastination and subjective wellbeing and academic performance, tells us that high academic procrastination leads to low subjective wellbeing and low academic performance not only directly, but also indirectly through increased cognitive test anxiety, which in turn leads to low subjective wellbeing and low academic performance. Thus, decreasing students' cognitive test anxiety through various self-regulated learning strategies and anxiety control techniques could reduce, in part, the negative effect of academic procrastination on subjective wellbeing and academic performance.

## 5 Conclusion

The recorded results marked the determining/influencing relationship of procrastination as a predictor of academic performance and perceived subjective state. We also identified a negative relationship between the level of evaluation anxiety and that of wellbeing.

Based on the analysis of results we can state that there is a need for educational intervention to control/reduce/eliminate academic procrastination. Disruptive behavior that generates stress as a result of evaluation anxiety inevitably leads to a decrease in wellbeing and the level of academic performance, which, as a consequence, calls for the learning of regulatory behavior. The study carried out unequivocally highlighted the fact that a high level of procrastination leads to a decrease in performance and subjective wellbeing both directly and indirectly, by increasing test anxiety, which in turn leads to a decrease in performance and subjective wellbeing. At the same time, as an element of specificity, the direct contribution of the study consists in understanding the interdependencies between procrastination - evaluation anxiety - wellbeing - school performance, the relationship of inverse proportionality between procrastination - evaluation anxiety and wellbeing - academic performance, in the sense that a high degree of anxiety and procrastination causes the recording of low levels of wellbeing and school performance In the context of sustainable education, we are in a position to emphasize the need for the responsible involvement of educational actors in controlling anxiety and procrastination behavior. The educational programs offered by specialists need to be doubled through the responsible involvement of each learner in their training so that the self-regulation of learning can be supported on an ever wider scale, regardless of whether we are talking about synchronous learning or asynchronous. Self-regulatory actions require careful attention to detail according to individual needs and interests, but without them, there is no chance of academic success and comfortable wellbeing. Achieving and maintaining wellbeing is a condition of sustainable development, which is an imperative of contemporary society. The study carried out confirms the need for responsible interventions at school/ university institutional level, but also for staff, teaching staff, and students alike.

Other successful educational mechanisms and strategies derived from specialized literature support the eradication of assessment anxiety by resorting to specific strategies such as: analyzing the levels of competence at the level of student groups, followed by an interactionist mediation (69); receiving positive feedback that has a buffering effect whereby post-negative arousal feedback remains low, relative to post-negative arousal feedback experienced among those who received negative feedback first (42); increasing perception students regarding their ability to cope with the stressful test situation (70); motivational strategies and techniques to bring students to a point of anxiety necessary for cognitive mobilization (71); addressing the most suitable assessment forms/tools, given that there are some studies confirming a strong relationship between test anxiety and the tool used; there was a statistically significant decrease in the mean anxiety score on the WTA scales for students administered the computer-based exam compared to the paper-based exam (2.48 vs. 3.90; *p* < 0.05) (72). It is suggested that both students' and teachers' technological knowledge/skills should be substantially improved to reduce test anxiety.

## 6 Limitations and implications of the study

Limitation of the study can be related to the selection of the sample of subjects. Given the fact that participation in the study was voluntary, the questionnaires being loaded in Google forms, there was an imbalance in the composition of the sample regarding gender differences (the female gender being the majority. For a better relevance of the research results, the size of the sample of subjects could be expanded not only at the national level, but also internationally, including regarding the level of studies, respectively bachelor's level and master's level, analyses will be carried out to highlight the registration of differences according to the level of studies.

Another aspect to point out is represented by the direct implications of the study, which are subsumed under the educational aspects and operate in the area of the necessary reduction of procrastination and evaluation anxiety in order to achieve favorable academic performance and a satisfactory state of wellbeing. Therefore, more extensive research oriented toward experimental interventions in the educational field could be enlightening on strategies that reduce procrastination and assessment anxiety in students.

Along the lines of future research directions, the approach of exploring and testing other mediator potentiators of evaluation anxiety and procrastination can be included. - For example, through a comparative analysis of the impact of online vs. traditional learning environments on procrastination and test anxiety, other mediators/facilitators can be identified, which once recognized can be removed through specific psychopedagogical interventions or through self-regulation. At the same time the role of cultural factors in the anxious behavior in the assessment could be investigated, as well as the role in the adoption of procrastination attitudes, especially since it is known that cultural factors can encourage both desirable and disruptive behavior.

Last but not least, interventions to support students and prevent procrastination should focus on programs that primarily increase the student's proactive attitude, planning skills, self-monitoring and effective time management, and awareness emotional and emotional regulation as a response in situations of stress and evaluation anxiety (73). At the same time, given the significant correlations between future time perspective, fatalistic and hedonistic present time perspective, and academic procrastination (74), it is recommended to implement various educational coaching strategies that activate future temporal orientation while decreasing fatalistic and hedonistic present temporal orientations.

## Data availability statement

The raw data supporting the conclusions of this article will be made available by the authors, without undue reservation.

## Ethics statement

The studies involving humans were approved by Scientific Council of Babes-Bolyai University. The studies were conducted in accordance with the local legislation and institutional requirements. The Ethics Committee/institutional review board waived the requirement of written informed consent for participation from the participants or the participants' legal guardians/next of kin because it was a personal option of the participants to participate as volunteer and the consent was gathered verbal under the ethical notice of the university.

## Author contributions

A-DM: Conceptualization, Investigation, Methodology, Writing – original draft. IA: Conceptualization, Data curation, Supervision, Validation, Writing – review & editing. A-VL: Formal analysis, Methodology, Validation, Writing – original draft. CS: Data curation, Formal analysis, Resources, Validation, Writing – review & editing.
